# Supplementary material for: Global turnover of soil mineral-associated and particulate organic carbon
Source: Nat Commun. 2024 Jun 22;15:5329. doi: 10.1038/s41467-024-49743-7 (PMC11193739; doi:10.1038/s41467-024-49743-7)
Supplement: Supplementary file 1 — Supplementary Information [file 41467_2024_49743_MOESM1_ESM.pdf]

Supplementary Information for
Global turnover of soil mineral-associated and particulate organic carbon
Zhenghu Zhou et al.

Supplementary Table 1 | Data sources of the covariates.

Covariates	Source	Resolution
Mean annual temperature and mean annual precipitation	WorldClim Version 2[1]	1 km × 1 km
Soil types, pH, clay plus silt content, base saturation, and cation exchange capacity	Harmonized World Soil Database Version 2.0[2]	1 km × 1 km
Elevation and slope	Openlandmap (https://openlandmap.org/)	250 m × 250 m
MODIS net primary productivity	EARTHDATA (https://ladsweb.modaps.eosdis.nasa.gov/)	500 m × 500 m
Nitrogen deposition	Global estimates of inorganic nitrogen deposition across four decades[3]	2° × 2.5°
Land cover	Terrestrial Ecoregions of the World[4] and the MODIS Land Cover product (http://files.ntsg.umt.edu/data/NTSG_Products/)	1 km × 1 km and 500 m × 500 m

1 Fick, S. E. & Hijmans, R. J. WorldClim 2: new 1-km spatial resolution climate surfaces for global land areas. *International Journal of Climatology* **37**, 4302-4315 (2017).
2 FAO & IIASA. *Harmonized World Soil Database version 2.0.* (2023).
3 Ackerman, D., Millet, D. & Chen, X. Global estimates of inorganic nitrogen deposition across four decades. *Global Biogeochemical Cycles* **33**, 100-107 (2019).
4 Olson, D. M. *et al.* Terrestrial Ecoregions of the World: A New Map of Life on Earth: A new global map of terrestrial ecoregions provides an innovative tool for conserving biodiversity. *BioScience* **51**, 933-938 (2001).

Supplementary Table 2 | The performance of six machine learning algorithms. *RMSE*, root mean square error. MAOC, mineral-associated organic carbon. POC, particulate organic carbon.

C Fractions	Machine learning algorithms	*RMSE*
MAOC	Random forest	0.041
	Extreme gradient boosting	0.047
	Support vector machine	0.060
	Recursive partitioning and regression tree	0.079
	Neural network	0.079
	Multivariable linear regression	0.079
POC	Random forest	0.056
	Extreme gradient boosting	0.063
	Support vector machine	0.073
	Recursive partitioning and regression tree	0.102
	Neural network	0.104
	Multivariable linear regression	0.102

Supplementary Table 3 | The prior range of the model parameters. MAOC, mineral-associated organic carbon. POC, particulate organic carbon.

Parameter Description	Symbol	Units	Minimum	Maximum	References
Decomposition rate of MAOC at 0–20 cm	KM_1	yr^{-1}	0.0001	0.2	Lavallee et al.[1]
Decomposition rate of POC at 0–20 cm	KP_1	yr^{-1}	0.002	0.8	Lavallee et al.[1]
The e-folding depth for carbon turnover of MAOC	z_M	cm	30	200	Koven et al.[2]
The e-folding depth for carbon turnover of POC	z_P	cm	30	200	Koven et al.[2]
Transfer coefficient from POC to MAOC	$T_{MAOCtoPOC}$	$kg\ m^{-2}\ (kg\ m^{-2})^{-1}$	0.05	0.8	Hararuk et al.[3]
Transfer coefficient from MAOC to POC	$T_{POCtoMAOC}$	$kg\ m^{-2}\ (kg\ m^{-2})^{-1}$	0.05	0.8	Hararuk et al.[3]
Diffusion rate	D	$kg\ m^{-2}\ (kg\ m^{-2})^{-1}\ yr^{-1}$	0	0.005	CLM[2] & ORCHIDEE-SOM[4]
Advection rate	A	$kg\ m^{-2}\ (kg\ m^{-2})^{-1}\ yr^{-1}$	0	0.005	CLM[2] & ORCHIDEE-SOM[4]

1 Lavallee, J. M., Soong, J. L., & Cotrufo, M. F. Conceptualizing soil organic matter into particulate and mineral-associated forms to address global change in the 21st century. *Global Change Biology 26*, 261-273 (2020).

2 Koven, C. D., Riley, W. J., Subin, Z. M., Tang, J. Y., Torn, M. S., Collins, W. D., Bonan, G. B., Lawrence, D. M. & Swenson, S. C. The effect of vertically resolved soil biogeochemistry and alternate soil C and N models on C dynamics of CLM4. Biogeosciences, 10, 7109-7131 (2013).

3 Hararuk, O., Xia, J., & Luo, Y. Evaluation and improvement of a global land model against soil carbon data using a Bayesian Markov chain Monte Carlo method. *Journal of Geophysical Research: Biogeosciences*, *119*, 403-417 (2014).

4 Camino-Serrano, M. et al. ORCHIDEE-SOM: modeling soil organic carbon (SOC) and dissolved organic carbon (DOC) dynamics along vertical soil profiles in Europe. *Geoscientific Model Development* 11, 937-957 (2018).

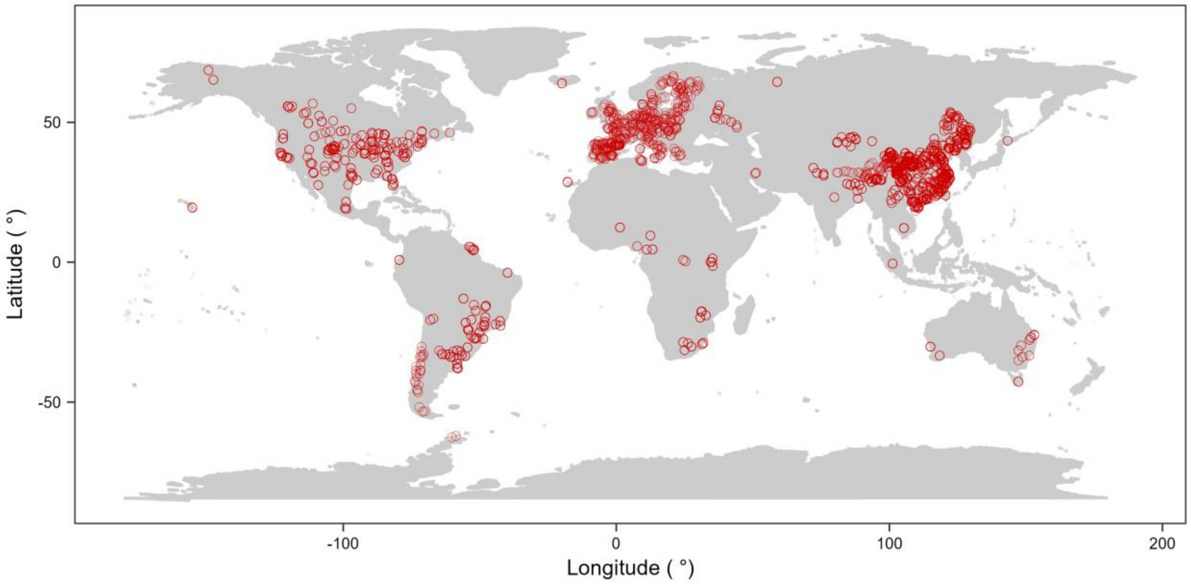

Supplementary Fig. 1 | The distribution of 8341 soil observations in this synthesis.

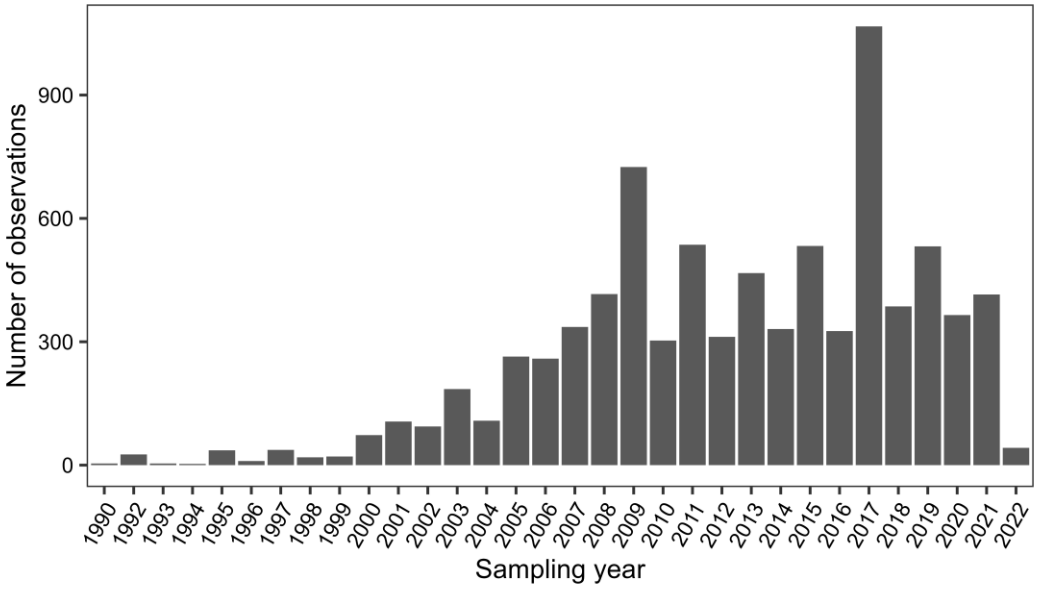

Supplementary Fig. 2 | The distribution of sampling year.

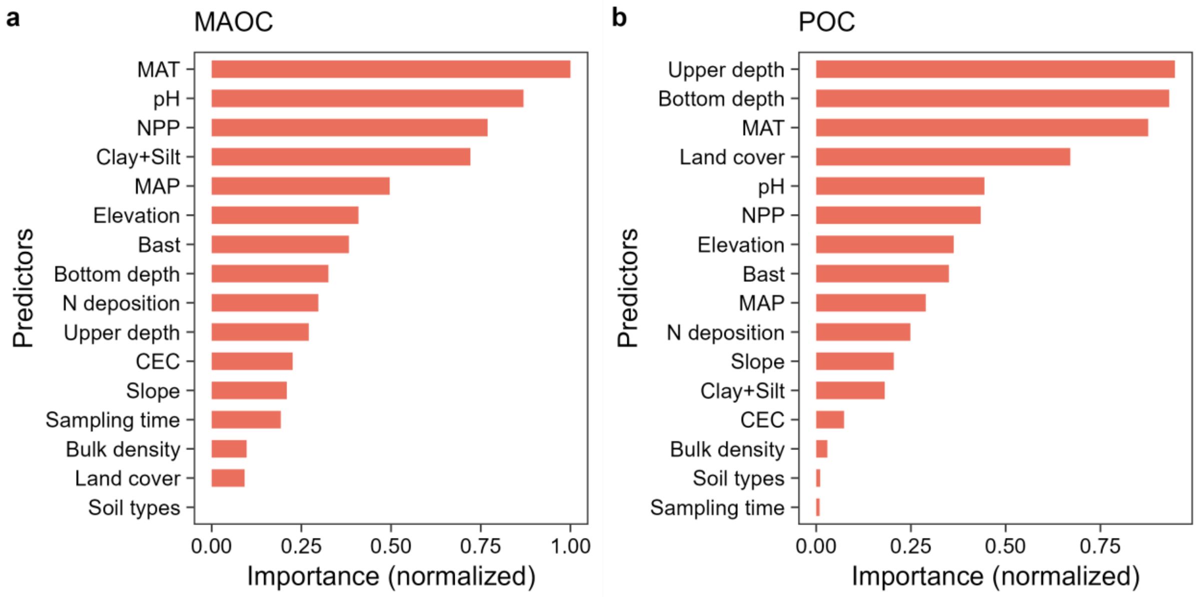

Supplementary Fig. 3 | The importance of predictors from full models. a The importance of predictors for mineral-associated organic carbon (MAOC). **b** The importance of predictors for particulate organic carbon (POC). Two types of importance (impurity and permutation) were first normalized to the interval of [0, 1] according to their maximum and minimum values. The mean of the normalized importance was then calculated. MAT, mean annual temperature; MAP, mean annual precipitation. NPP, net primary productivity. CEC, cation exchange capacity. Bast, base saturation.

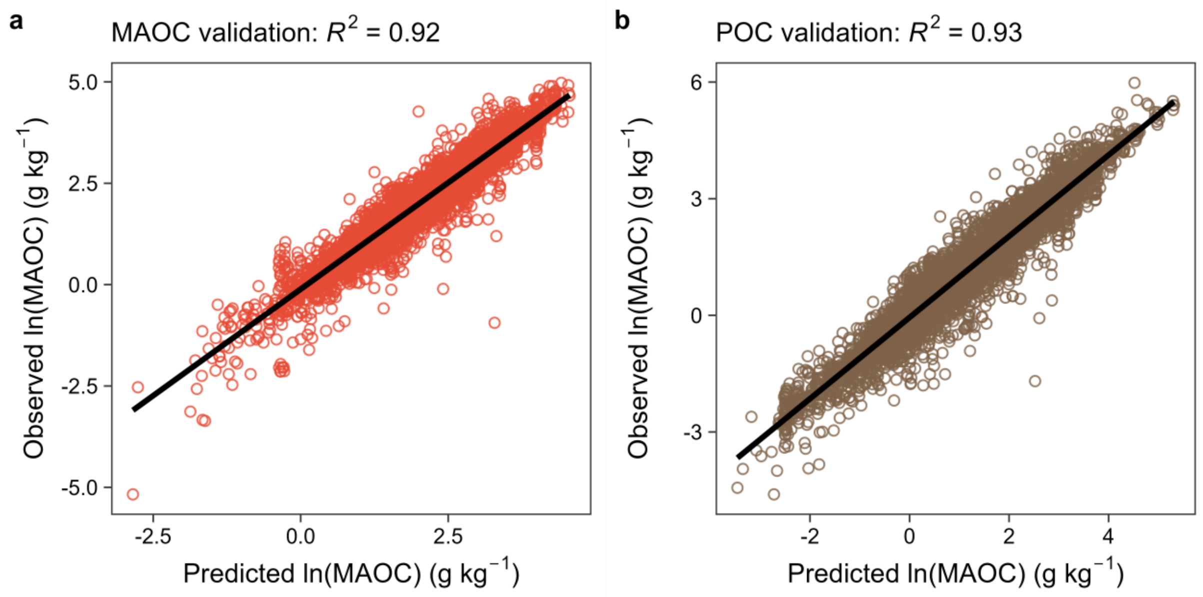

Supplementary Fig. 4 | The *K*-fold cross validation of random forest models. a The K-fold cross validation for mineral-associated organic carbon (MAOC). **b** The K-fold cross validation for particulate organic carbon (POC). ln, natural logarithmic transformation.

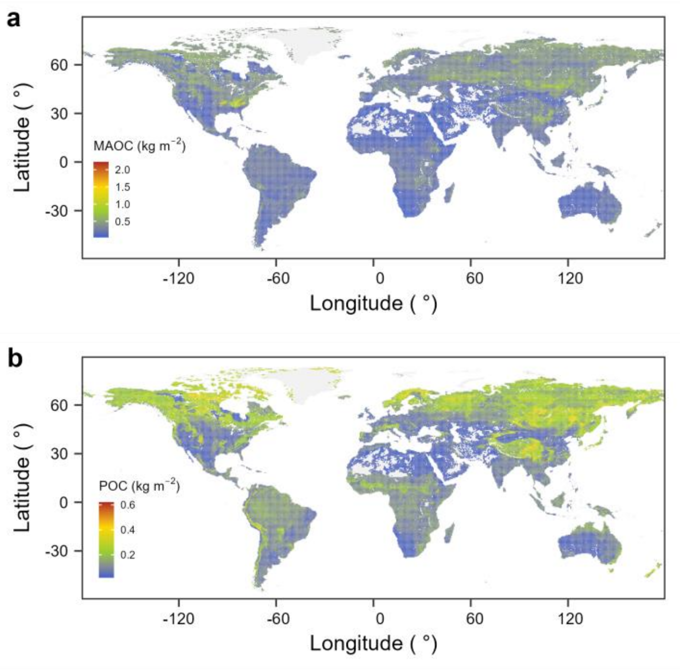

Supplementary Fig. 5 | Uncertainties of current estimations of mineral-associated and particulate organic carbon. a Uncertainties of current estimations of mineral-associated organic carbon (MAOC). **b** Uncertainties of current estimations of particulate organic carbon (POC). The uncertainty is standard deviation from the 100-time bootstrapping.

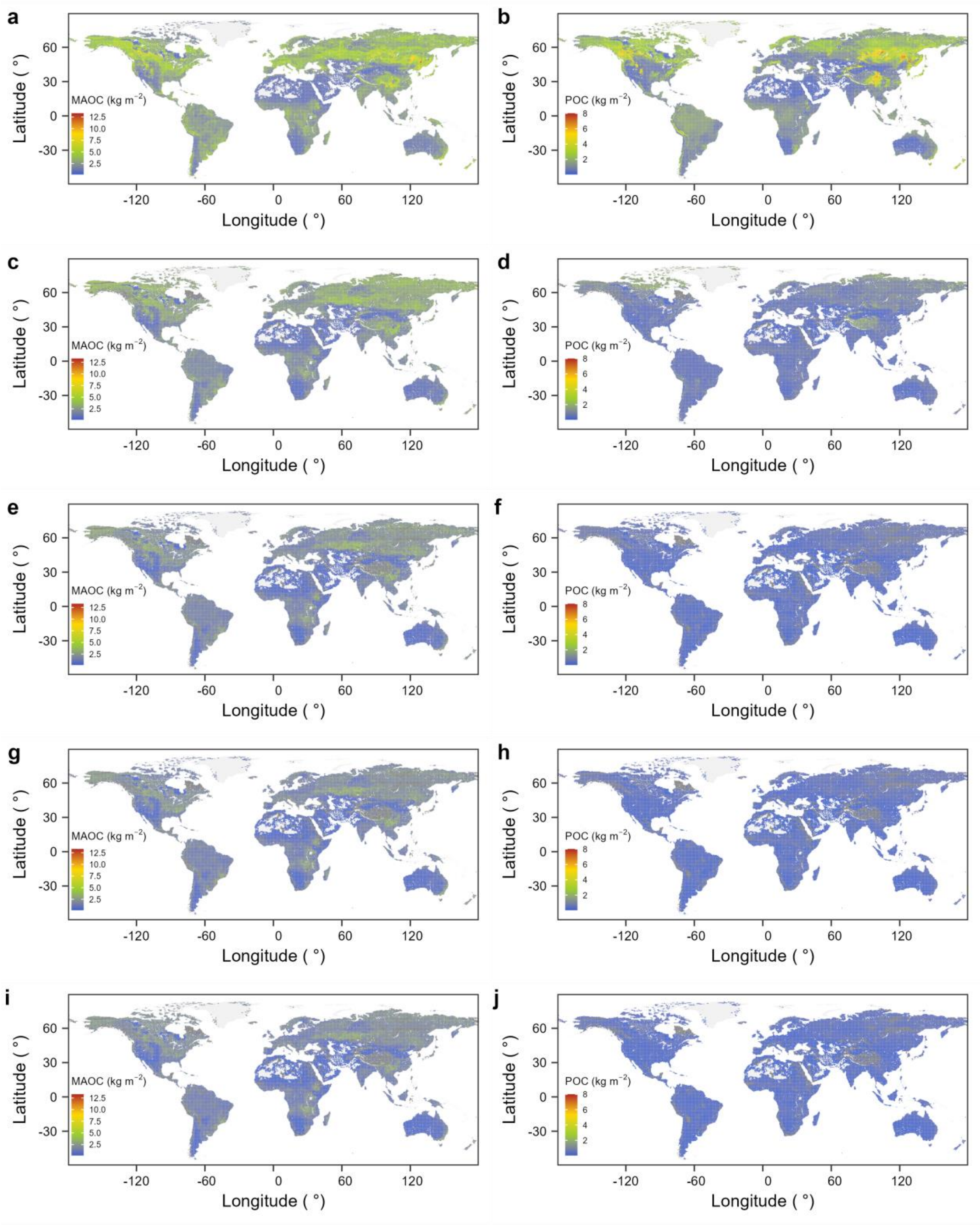

Supplementary Fig. 6 | Global estimations of mineral-associated and particulate organic carbon by soil layers. **a** and **b** 0–20 cm. **c** and **d** 20–40 cm. **e** and **f** 40–60 cm. **g** and **h** 60–80 cm. **i** and **j** 80–100 cm. MAOC, mineral-associated organic carbon. POC, particulate organic carbon.

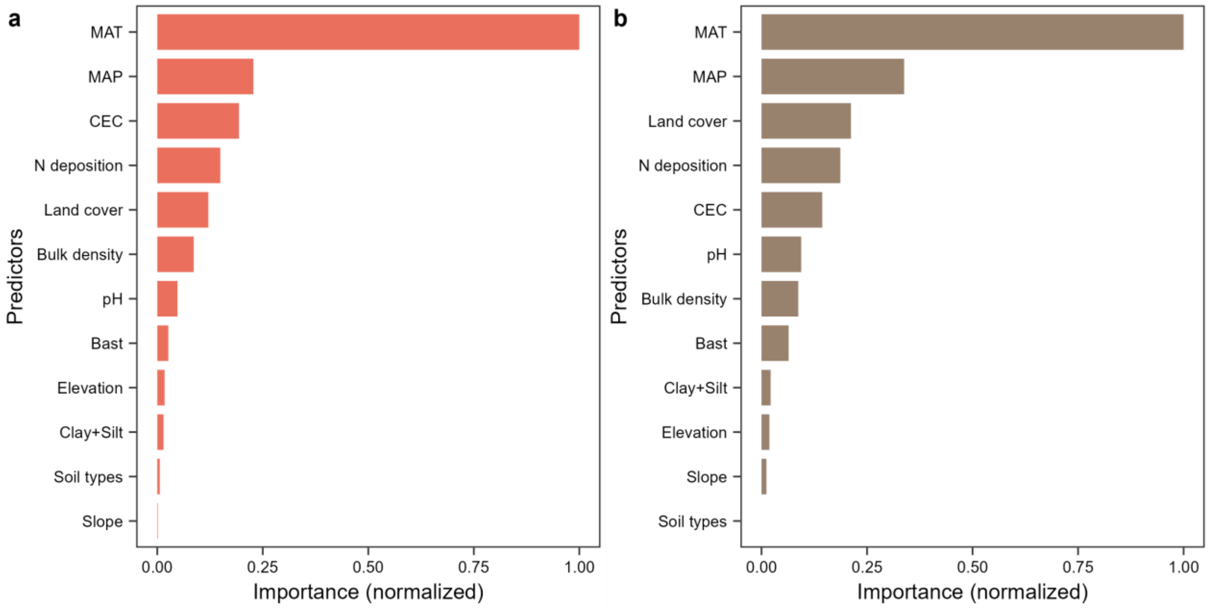

Supplementary Fig. 7 | The importance of predictors for mineral-associated and particulate organic carbon turnovers. a The importance of predictors for mineral-associated organic carbon (MAOC) turnovers. **b** The importance of predictors for particulate organic carbon (POC) turnovers. Two types of importance (impurity and permutation) were first normalized to the interval of [0, 1] according to their maximum and minimum values. The mean of the normalized importance was then calculated. MAT, mean annual temperature; MAP, mean annual precipitation. NPP, net primary productivity.

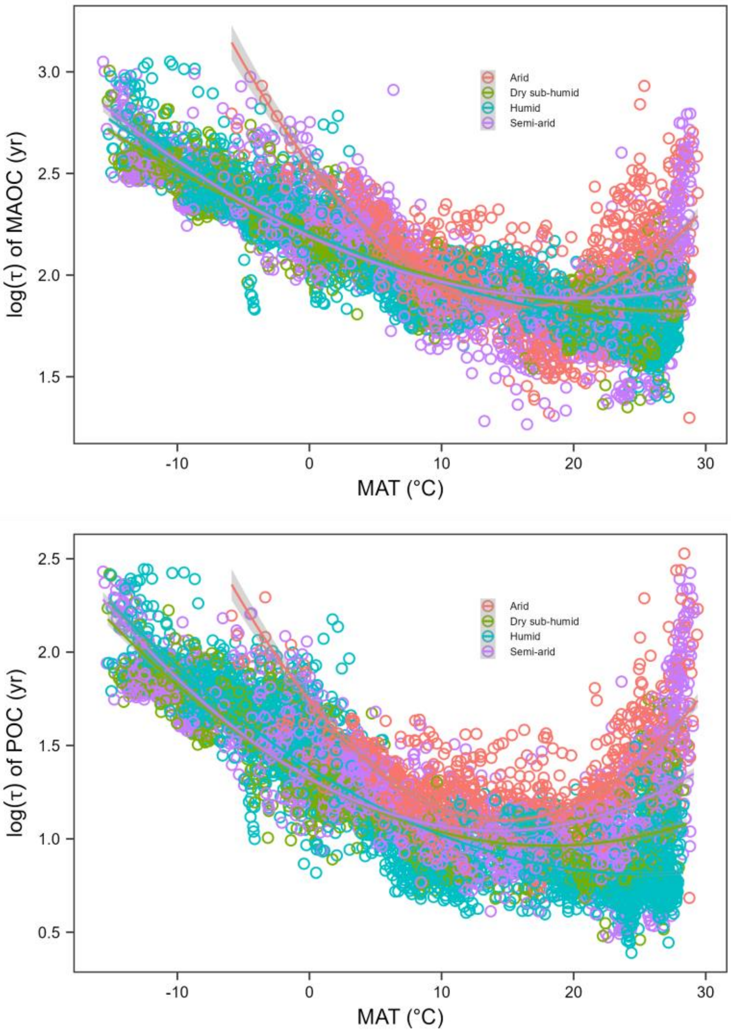

Supplementary Fig. 8 | Temperature dependent turnover times vary with aridity. τ, turnover time. MAOC, mineral-associated organic carbon. POC, particulate organic carbon. MAT, mean annual temperature.

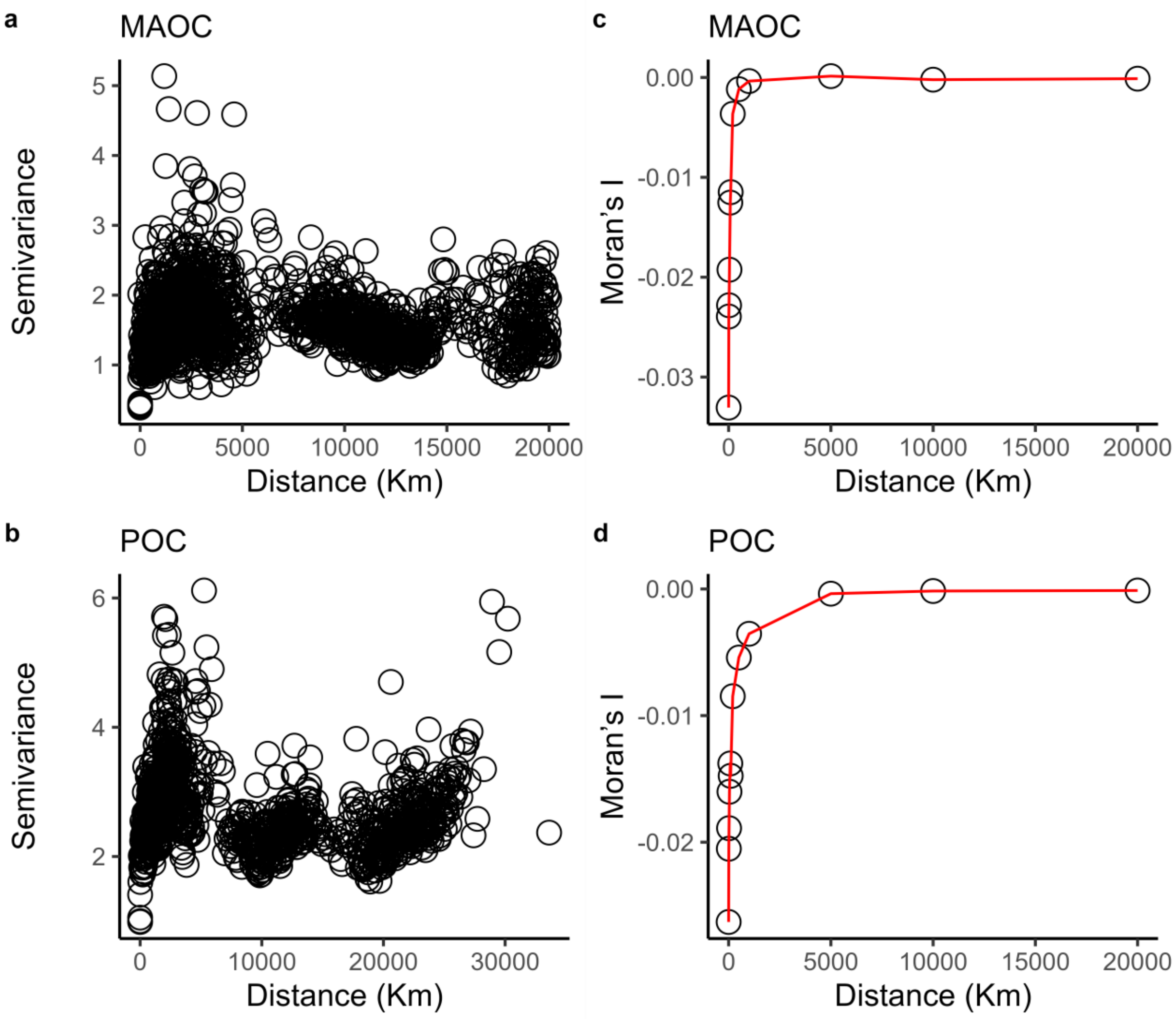

Supplementary Fig. 9 | Results of spatial auto-correlation testing. a and **b** Semivariogram showing the spatial auto-correlation for mineral-associated (MAOC) and particulate organic carbon (POC), respectively. **c** and **d** Moran's index of the random forest models for MAOC and POC, respectively. Greater significantly positive Moran's index indicates that there is spatial auto-correlation for the given variable and distance threshold, and we did not find the significant spatial auto-correlation.

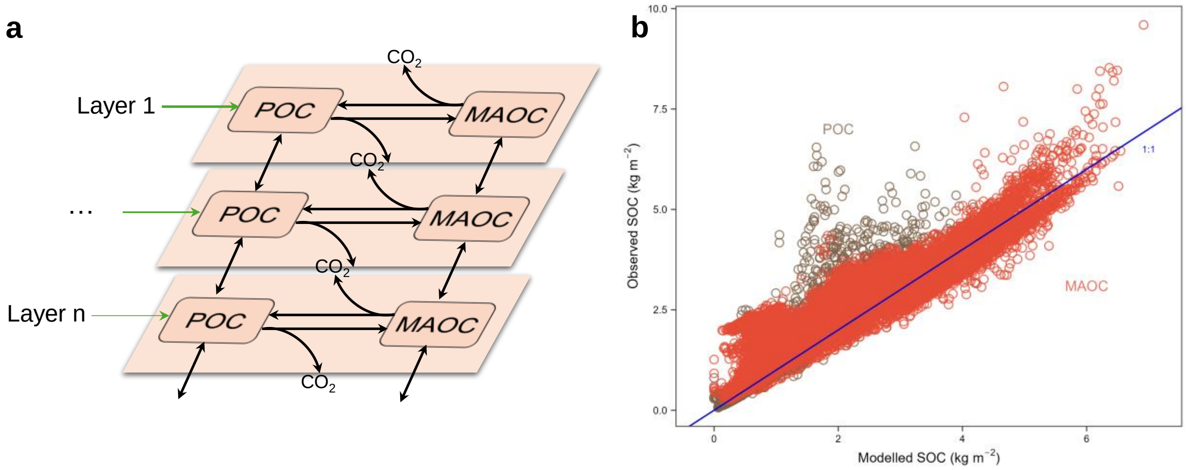

Supplementary Fig. 10 | Model scheme and validation. a mineral-associated (MAOC) and particulate organic carbon (POC) dynamics model. **b** The relationship between observed and modelled soil organic carbon (SOC).

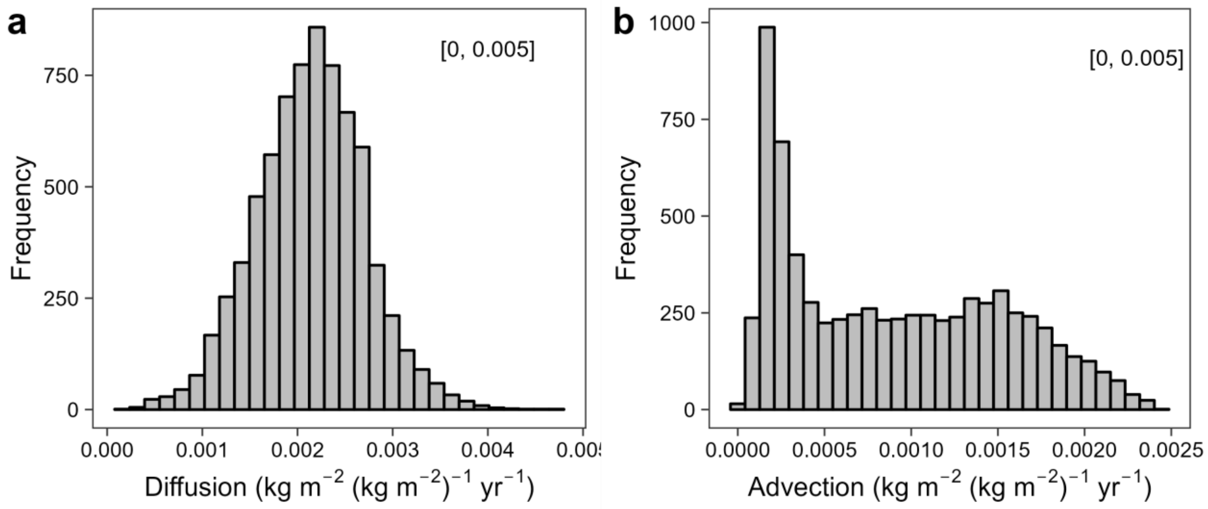

Supplementary Fig. 11 | Distributions of the optimized diffusion and advection. a Distribution of the optimized diffusion**. b** Distribution of the optimized advection
